# Sex differences in airway volume and 3-dimensional shape in Japanese adults

**DOI:** 10.1038/s41598-023-41263-6

**Published:** 2023-08-25

**Authors:** Chihiro Tanikawa, Ayaka Oka, Yuki Shiraishi, Takashi Yamashiro

**Affiliations:** Department of Orthodontics and Dentofacial Orthopedics, Graduate School of Dentistry, Osaka University, 1-8 Yamadaoka, Suita, Osaka 565-0871 Japan

**Keywords:** Dentistry, Oral anatomy, Health care, Three-dimensional imaging

## Abstract

(1) To establish normative data for three-dimensional (3D) measurements of the upper airway in young Japanese adults, and (2) to investigate sex-related differences in linear and volumetric measurements, as well as shape. This study employed cone-beam computed tomography (CBCT) images of 56 Japanese young adults preselected from among 1000 patients, so that samples matched a historic 2D cephalometric cohort with normal occlusion using propensity score matching. Three-dimensional models of the oropharynx and hypopharynx were reconstructed from CBCT images and their volumes were calculated. We defined 20 landmarks on the surface of the 3D model and performed seven linear measurements between them. The mean and standard deviation of the linear measurements were calculated as the normative data for each sex as well as the volumes. Sex-related differences were analyzed using *t*-test (*p* < 0.05). Principal component discriminant analysis of the coordinate values of the landmarks was also performed to examine sex differences in shape. The normative ranges of the 3D measurements of the oropharynx and hypopharynx were determined according to sex. Sex-related differences in the measurement results were observed in hypopharyngeal length but not in volume. The hypopharynx length in males was significantly longer than that in females. The discriminant analysis showed that males tended to show longer and straight shapes, while females showed inversed triangular shapes from the frontal view. This result will allow clinicians to evaluate how patient airway characteristics differ from the normative 3D morphology of the upper airway.

## Introduction

Obstructive sleep apnea (OSA) can be related not only to the patient’s primary craniofacial disharmony (e.g., micrognathia, retrognathism, shorter cranial base, and steep mandibular plane angle)^1^, but may also be related to the secondary repositioning of the maxilla/mandible through orthognathic surgery because it modifies the shape of the upper airway. For example, mandibular setback can narrow the pharyngeal airway by the posterior movement of the hyoid bone, while mandibular set-forward can increase the space^2,3^. Thus, evaluation of the upper airway and screening of patients who have risk factors for OSA are essential for orthodontists when determining orthodontic plans.

Recently, the evaluation of the three-dimensional (3D) upper airway using cone beam computed tomography (CBCT) has received attention because of its potential to prevent the possible adverse effects of orthognathic surgery^4^. CBCT can be used to evaluate airway dimensions more comprehensively than traditional 2D radiographs^5^. Previous researchers have reported the measurement of variables such as airway volume and cross-sectional area using CBCT, with moderate to excellent reliability^4,5^. However, almost all these reports have focused on the 3D volume of the upper airway. It is obvious that the same volume can exhibit several shapes, which is hypothesized to be important for OSA. In fact, a computational fluid dynamics analysis showed that the pharyngeal airway shape in children with OSA significantly affected the internal pressure distribution in comparison to nasal resistance. The model may also help explain regional dynamic airway narrowing during expiration^6^. Further, when orthodontists plan orthodontic treatment in combination with orthognathic surgery, the normative data of the 3D morphology of the upper airway in young adults would be useful for understanding the patient’s airway characteristics, which might reduce the adverse effects of orthognathic surgery on the airway.

Ethical issues prevent CBCT images from being obtained in cases of normal occlusion. To solve this problem, a previous study^7^ sampled patients who underwent CBCT for non-skeletal problems (e.g., impacted teeth) in private orthodontic clinics using a propensity score to match these patients with a historic cohort in a report that included cephalograms of individuals with normal occlusion. A previous study also calculated the optimal sample size (n = 56) to represent the population corresponding to individuals with normal occlusion based on a power analysis with the ANB angle from a total of approximately 1000 patients^7^.

Thus, we aimed to establish normative data for 3D measurements of the upper airway in young Japanese adults according to sex. Furthermore, we investigated sex-related differences in linear and volumetric measurements as well as their shape.

## Materials and methods

Ethical approval for this study was obtained from the Institutional Review Board of Osaka University Dental Hospital (No. H30-E5-1).

### Subjects

The samples of this retrospective study were recruited from among 1000 patients who underwent diagnostic cone-beam CT (CBCT) for non-routine and non-skeletal orthodontic diagnoses, such as impacted teeth, at a private dental clinic between 2000 and 2015, which were used in a previous study that aimed to establish 3D cephalometric norms in Japanese^7^. To minimize radiation exposure, scans were only performed when the diagnostic benefits outweighed the risks associated with radiation exposure. The inclusion criteria were as follows: 17–40 years of age; skeletal 1 malocclusion (ANB = 0–5 degrees; ANB indicates the sagittal skeletal relationship of the maxilla and mandible in the cephalometric analysis). The exclusion criteria were as follows: history of orthodontic treatment, craniofacial or growth abnormalities, systemic disease, or temporomandibular joint disorder. Data selection was conducted using the propensity score matching method, which allows for the selection of patients that are matched to a 2D historic cohort^8^ regarding the ANB and Frankfurt-mandibular plane angle (FMA). CBCT data from 56 patients (male, n = 28; mean age, 22.95 ± 5.97 years; ANB = 3.04 ± 1.36 degrees; FMA = 29.07 ± 5.76 degrees; women, n = 28, mean age, 24.68 ± 4.28 years; ANB = 3.14 ± 1.25 degrees; FMA = 29.54 ± 5.06 degrees) were employed in the present study.

### CBCT image processing

Patients were instructed to place their jaw with maximum intercuspation and rest their lips and tongue. The Frankfurt horizontal (FH) plane of the patients was parallel to the floor. Patients were asked to breathe normally through their nose without swallowing and to avoid moving their head or tongue during the scanning process. CBCT was conducted using an Alphard-3030 in the low-dose mode (Asahi Roentgen Ind. Co., Ltd., Kyoto, Japan) at 80 kV and 2 mA, with a 1-voxel size of 0.39 mm^3^. The field of view was 20 cm × 20 cm. CBCT images were saved in the DICOM format. The time required for imaging was 15 s. The subject's posture during the imaging was standing.

### Segmentation and measurement

The surface of the airway was generated using ITK-SNAP (National Library of Medicine and National Institutes of Health, Bethesda, MD, USA). ITK-SNAP can generate STL files from DICOM data semi-automatically, based on a previously reported method^7^.

### Definitions of anatomical boundary and volume calculation

**Figure 1:**
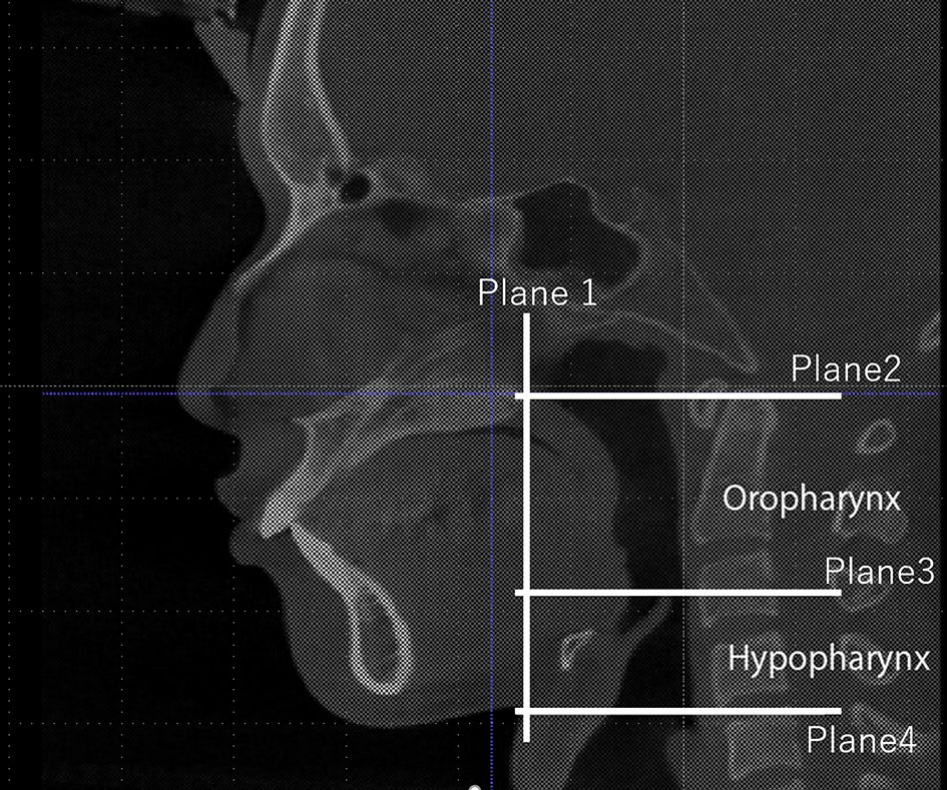
Definition of the oropharynx and hypopharynx. The anterior boundary was the vertical plane passing through the posterior nasal spine (plane 1); the lateral and posterior boundaries consisted of the pharyngeal walls, the anterior boundary, the anterior wall of the pharynx, the base of the tongue, and the soft palate; the superior boundary of the oropharynx was the plane parallel to the FH plane through the posterior nasal spine (PNS) (plane 2); the plane parallel to the FH plane through the tip of the epiglottis (plane 3) was used to separate the oropharynx and hypopharynx; the inferior boundary of hypopharynx was the plane parallel to the FH plane through the most inferior and anterior points on the corpus of the fourth cervical vertebra (plane 4).

The anatomical boundaries of the oropharynx and hypopharynx were defined as follows (Fig. 1). Vertically, the superior boundary of the oropharynx is defined as the plane parallel to the FH plane through the posterior nasal spine. A plane parallel to the FH plane through the tip of the epiglottis was used to separate the oropharynx and hypopharynx. The inferior boundary of the hypopharynx was defined as the plane parallel to the FH plane through the most inferior and anterior points on the fourth cervical vertebra corpus.

The volumes of the oropharynx and hypopharynx were calculated.

### Identification of landmarks and data processing

**Figure 2:**
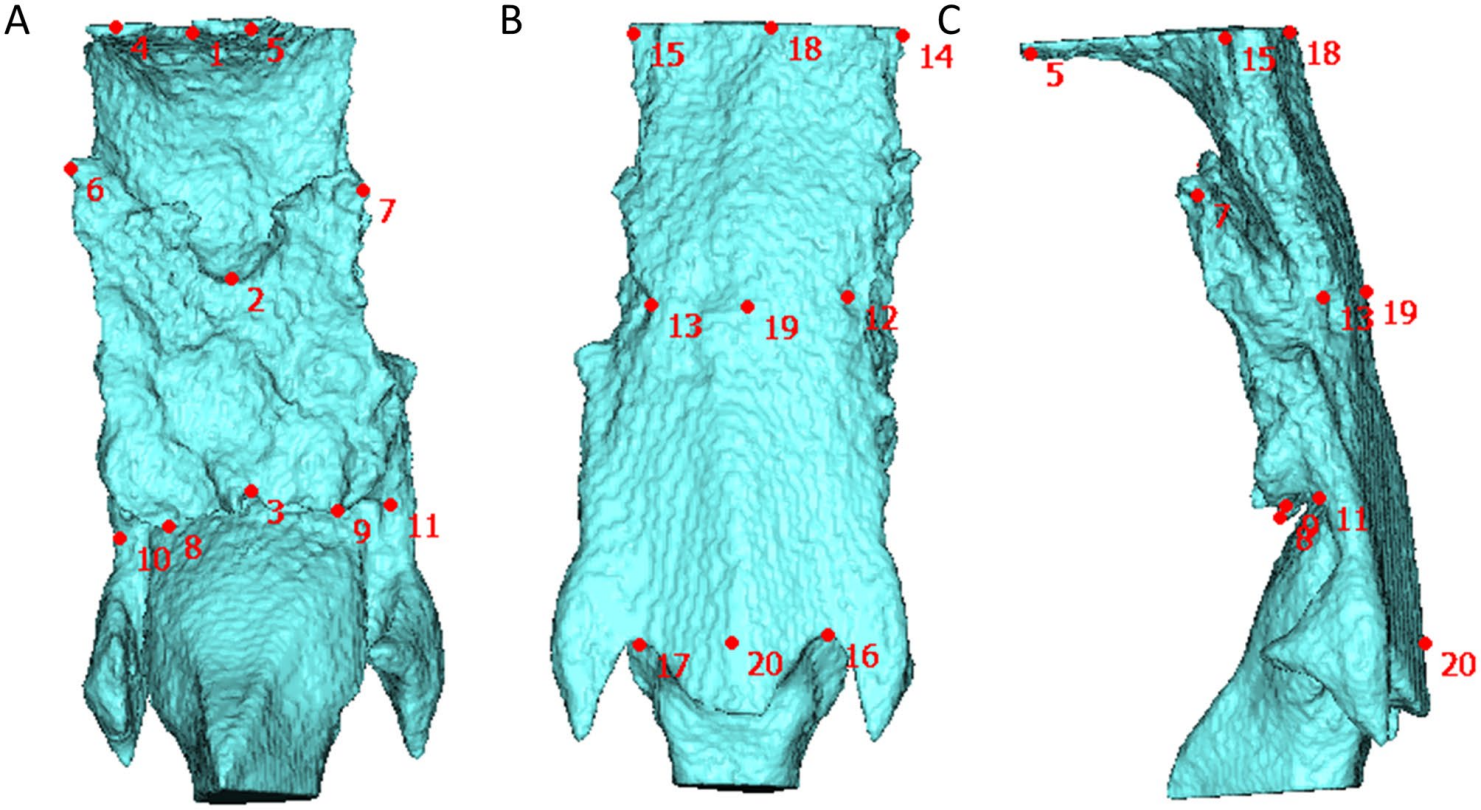
Definition of 20 landmarks of the oropharynx and hypopharynx. (**A**) Frontal view; (**B**) backward view; (**C**) lateral view from the left.

The positions of the 20 landmarks of the oropharynx and hypopharynx (Fig. 2 and Table 1) were identified by visual inspection of the image and digitized using a computer mouse cursor and HBM-Rugle (Medic Engineering Co., Kyoto, Japan). Seven linear measurements were performed between reference points as follows, P1–P2, P1–P3, P12–P13, P14–P15, P16–P17, and P18–P19. These measurements were arbitrarily selected by the authors to represent the shapes of the oropharynx and hypopharynx.

**Table 1:** Definition of 20 landmarks of the oropharynx and hypopharynx.

Landmark	Definition
P1	Posterior nasal spine (PNS)
P2	Tip of uvula (UT)
P3	Most superior point of vallecula of epiglottis
P4	Deepest posterior margin of palatine bone (right)
P5	Deepest posterior margin of palatine bone (left)
P6	Most lateral point of the oropharyngeal wall on the right side
P7	Most lateral point of the oropharyngeal wall on the left side
P8	Most inferior point of vallecula of epiglottis on the right side
P9	Most inferior point of vallecula of epiglottis on the left side
P10	Most posterior point of the epiglottis on the right side
P11	Most posterior point of the epiglottis on the right side
P12	Narrowest point of the oropharynx on the right side
P13	Narrowest point on the left side
P14	Most superior and lateral point on the right side
P15	Most superior and lateral point on the left side
P16	Most anterior point of the arytenoid cartilage on the right side
P17	Most anterior point of the arytenoid cartilage on the left side
P18	Middle point of P14 and P15
P19	Middle point of P12 and P13
P20	Middle point of P16 and P17

### Intra-observer reproducibility for the volume, landmarks, and distances

Twelve CBCT data points were randomly selected from the total data. One of the authors (A.O.) conducted the above measurements (i.e., volume measurements and landmark identification) twice with a 1-week interval to confirm inter-observer reproducibility. Furthermore, another author (Y.S.) conducted the same measurements to determine intra-observer reproducibility. Intra-correlation coefficients (ICC) were calculated. “Moderate” and “substantial” agreement was defined as ICC > 0.40 to ≤ 0.60 and 0.60 to ≤ 0.80, respectively, while ICC ≥ 0.81 indicated an “almost perfect” agreement.

### Sex differences

#### Comparison of distances and airway volume

Sex differences were tested using *t*-test for volume and linear measurements. Furthermore, the mean airway shape was represented as a diagram connecting the mean coordinates of the landmarks for each sex.

#### The principal discriminant analysis of shape

To describe the variations in the airway morphology among skeletal 1 subjects and examine the differences in landmark coordinates between sex groups, the following calculation was performed. First, Procrustes registration, a statistical shape analysis (i.e., geometric morphometric analysis) used to assess the distribution of a set of shapes by translation, uniform scaling, and rotation, was performed. Second, the dimensionality of the landmark data was reduced by performing a principal component analysis (PCA) for the coordinates of the landmarks. Principal components (PCs) with variances greater than 10% of the total variance were used. Finally, to examine sex shape differences in terms of the aforementioned PCs, PCs were entered into a discriminant analysis to discriminate between male and female shapes, as follows:

$$ \left[ {{\text{Male}}\;(1)\;{\text{or Female}}\;{(}- {1)}} \right] = {\text{w}}1 \, \times {\text{PC}}1 \, + {\text{w}}2 \, \times {\text{PC}}2 + \cdots + {\text{wk }} \times {\text{PCk}} $$

where k indicates the number of PCs; w1, w2,…, wk indicate the coefficient values of the discriminant regression analysis; and PCs 1, 2, …, k are dependent variables. Statistical significance was set at p ≤ 0.05. The correctly classified percentage of the total samples was calculated when 0 was set as the threshold value for discriminant results.

All calculations were conducted using MATLAB (2021a, MathWorks, Natick, MA, USA).

### Ethics approval and consent to participate

All methods were performed in accordance with relevant guidelines and regulations. All experimental protocols were approved by the Research Ethics Committee of Osaka University Dental Hospital (No. H30-E5-1). Informed consent was obtained from all patients and/or their legal guardians using the opt-out method.

## Results

### Reproducibility of volume measurements and the landmark identification

The inter- and intra-observer reliabilities showed “almost perfect agreement” (Table 2). All landmarks, except P4 and P5, showed “almost perfect agreement” for both inter-observer and intra-observer reproducibility (Table 3). P4 and P5 show “substantial agreement” on the vertical axis. Therefore, these two landmarks were eliminated from the following distance analysis.

**Table 2:** Inter- and intra-observer reproductivity of volume segmentation (intra-class coefficient).

	Difference (mm^3^)			95% CI for the difference		ICC	
	Mean	SD	SE	Inferior limit	Superior limit	r	p-value
Inter-observer							
Oropharyngeal volume	3.3	224.4	62.2	132.4	− 135.6	1.00	3.80E−29
Hypopharyngeal volume	12.7	159.5	46.0	88.6	− 114.1	1.00	1.60E−18
Total pharyngeal volume	14.5	293.7	84.8	172.1	− 201.1	1.00	1.60E−18
Intra-observer							
Oropharyngeal volume	91.3	513.8	148.3	417.7	− 235.2	0.99	2.30E−12
Hypopharyngeal volume	243.0	299.5	86.5	− 52.7	− 433.3	1.00	7.90E−14
Total pharyngeal volume	151.7	548.5	158.3	196.8	− 500.2	1.00	1.60E−14

*SD* standard deviation, *SE* standard error, *CI* confidence interval, *ICC* intra-class coefficient.

**Table 3:** Inter-observer and intra-observer reproductivity of landmark identification (intra-class coefficient).

Landmark	Inter-observer			Intra-observer		
	x	y	z	x	y	z
P1	0.99	0.99	0.97	1.00	1.00	1.00
P2	0.92	1.00	1.00	0.92	0.98	1.00
P3	0.99	1.00	0.99	1.00	1.00	1.00
P4	0.95	0.58	0.99	0.99	0.81	1.00
P5	0.89	0.59	1.00	0.99	0.79	1.00
P6	0.99	1.00	1.00	1.00	1.00	1.00
P7	1.00	1.00	0.9	1.00	1.00	1.00
P8	0.97	1.00	0.99	0.99	1.00	1.00
P9	0.99	1.00	0.93	0.98	1.00	1.00
P10	0.96	0.96	1.00	0.99	0.99	1.00
P11	0.90	0.95	1.00	0.99	0.99	1.00
P12	0.95	0.8	0.93	0.99	0.98	0.99
P13	1.00	0.84	0.98	0.98	0.98	0.99
P14	1.00	0.99	1.00	1.00	1.00	1.00
P15	1.00	0.98	0.89	1.00	1.00	0.88
P16	0.97	1.00	1.00	0.99	1.00	1.00
P17	0.97	1.00	1.00	0.99	1.00	1.00
P18	0.92	0.99	0.98	0.85	1.00	1.00
P19	0.96	0.81	0.96	0.96	0.99	1.00
P20	0.97	1.00	1.00	0.99	1.00	1.00

p < 0.05 was confirmed for all values.

### Sex differences in volume and distances

There were no significant differences in the volumes of the oropharynx and hypopharynx between sexes (Table 4). The coefficient of variation of the volumes ranged from 22 to 43%, indicating that the volumes vary among individuals, especially in the hypopharynx (41–43%). Compared to males, females showed a 12% smaller coefficient of variation in the oropharynx, indicating that males had greater volume variation in the oropharynx than females.

**Table 4:** Sex differences in the segmented volume of the upper airway.

Volume (mm^3^)		Mean	SD	Coefficient of variation (%)	Min	Max	Median	p-value
Oropharyngeal volume	Total	16,415.3	5086.7	31	6669.7	36,568.3	16,047.8	0.13
	Male	16,984.3	6319.0	37	8432.0	36,568.3	16,047.8	
	Female	15,865.3	3544.0	22	6669.7	24,196.3	16,095.4	
Hypopharyngeal volume	Total	8811.1	3757.2	43	2645.2	19,636.7	8384.0	0.41
	Male	9643.1	4036.9	42	4077.2	19,712.8	9108.0	
	Female	8145.1	3376.4	41	2411.9	15,039.1	7740.6	
Total pharyngeal volume	Total	25,296.7	7811.7	31	9397.3	56,281.0	24,869.0	0.21
	Male	26,627.5	9451.2	35	13,998.5	56,281.0	24,745.5	
	Female	24,010.4	5684.8	24	9397.3	35,463.2	25,180.9	

*SD* standard deviation, *Min* minimum, *Max* maximum.

**Figure 3:**
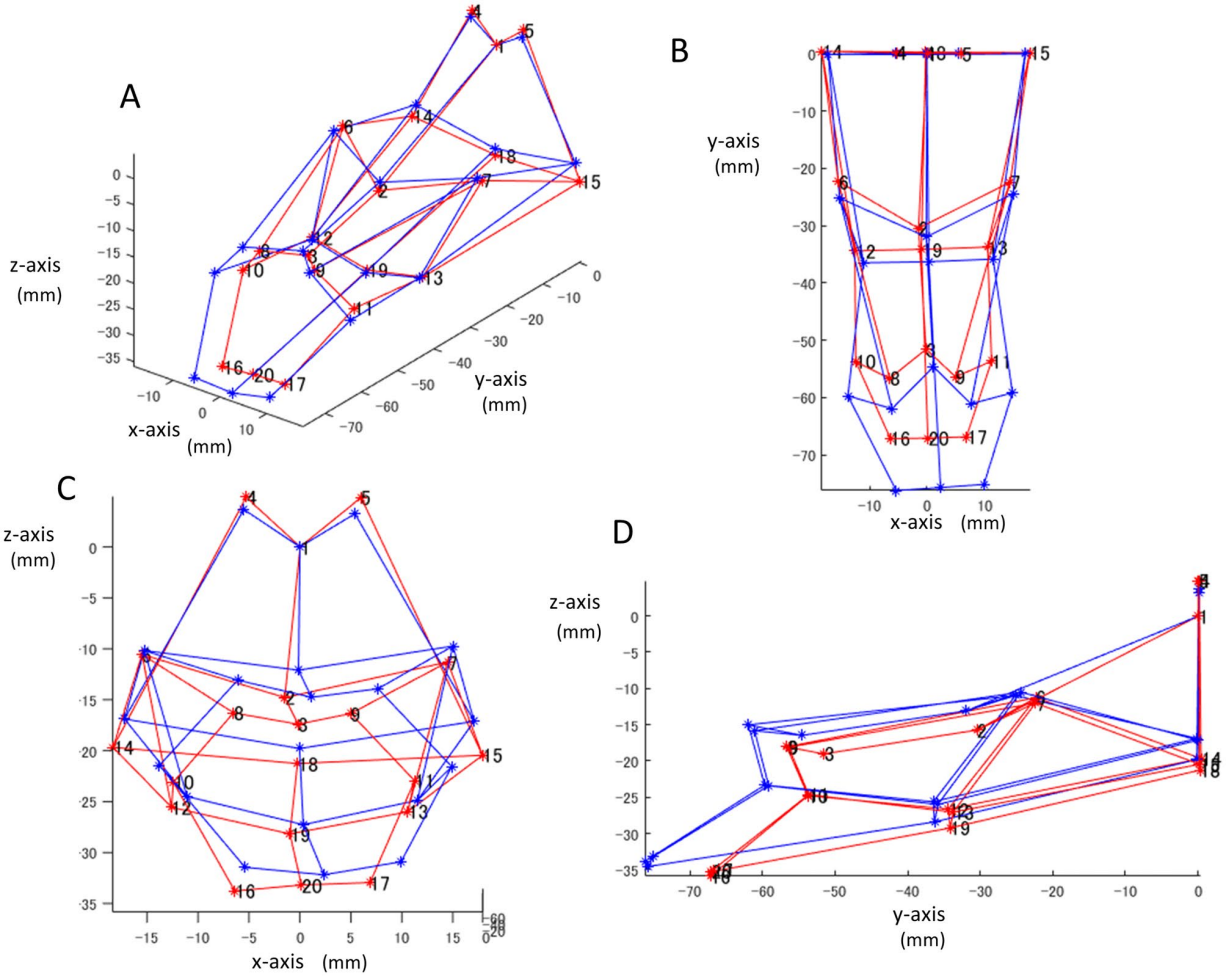
The mean airway shape in each group. Red indicates female. Blue indicates male. (**A**) Oblique view. (**B**) Frontal view. (**C**) Bottom view. (**D**) Lateral view (left side). The numbers represent landmark numbers (P1–P20; Table 1).

The mean airway shape in each sex group is shown in Fig. 3. The comparison of the distances showed that P3–P20 and P19–P20 were smaller in females than in males (p = 0.000, Table 5). This means that the distance between the most superior point of the vallecula of the epiglottis (P3) and the most anterior point of the arytenoid cartilage (P20) was smaller in females. Furthermore, the narrowest point of the oropharynx (P19) and the most anterior point of the arytenoid cartilage (P20) were smaller in females than in males while the other distances showed no differences. This indicates that the distance between the narrowest point and the bottom of the hypopharynx is smaller in females. As for the transverse distances, there were no significant differences between the sex groups, including the narrowest distances.

**Table 5:** Sex differences in the distances between landmarks.

Distance (mm)	Male				Female				p-value
	Mean	Min	Max	SD	Mean	Min	Max	SD	
P1–P2	34.87	23.38	48.62	5.90	34.57	26.89	43.01	3.80	0.828
P1–P3	57.38	44.77	71.04	6.73	55.16	40.10	67.05	6.40	0.220
P12–P13	22.90	15.16	36.90	5.15	23.40	17.77	29.81	3.47	0.676
P14–P15	34.41	21.60	46.16	6.17	36.78	24.43	49.67	6.10	0.162
P16–P17	15.72	3.90	24.59	6.06	13.43	4.60	23.03	4.40	0.118
P18–P19	37.45	26.76	58.01	7.06	35.51	28.55	48.94	4.85	0.245
P19–P20	40.43	28.56	59.68	6.85	33.84	25.27	42.66	5.29	0.000*
P2–P19	15.45	6.42	32.80	5.61	13.63	5.35	19.17	3.07	0.163
P3–P19	22.99	11.72	32.29	5.47	20.78	9.73	28.88	4.60	0.128
P3–P20	28.87	20.28	43.44	5.04	23.42	17.66	30.54	3.02	0.000*

*SD* standard deviation, *Min* minimum, *Max* maximum. *p = 0.05.

### Sex difference in the shape analysis

**Figure 4:**
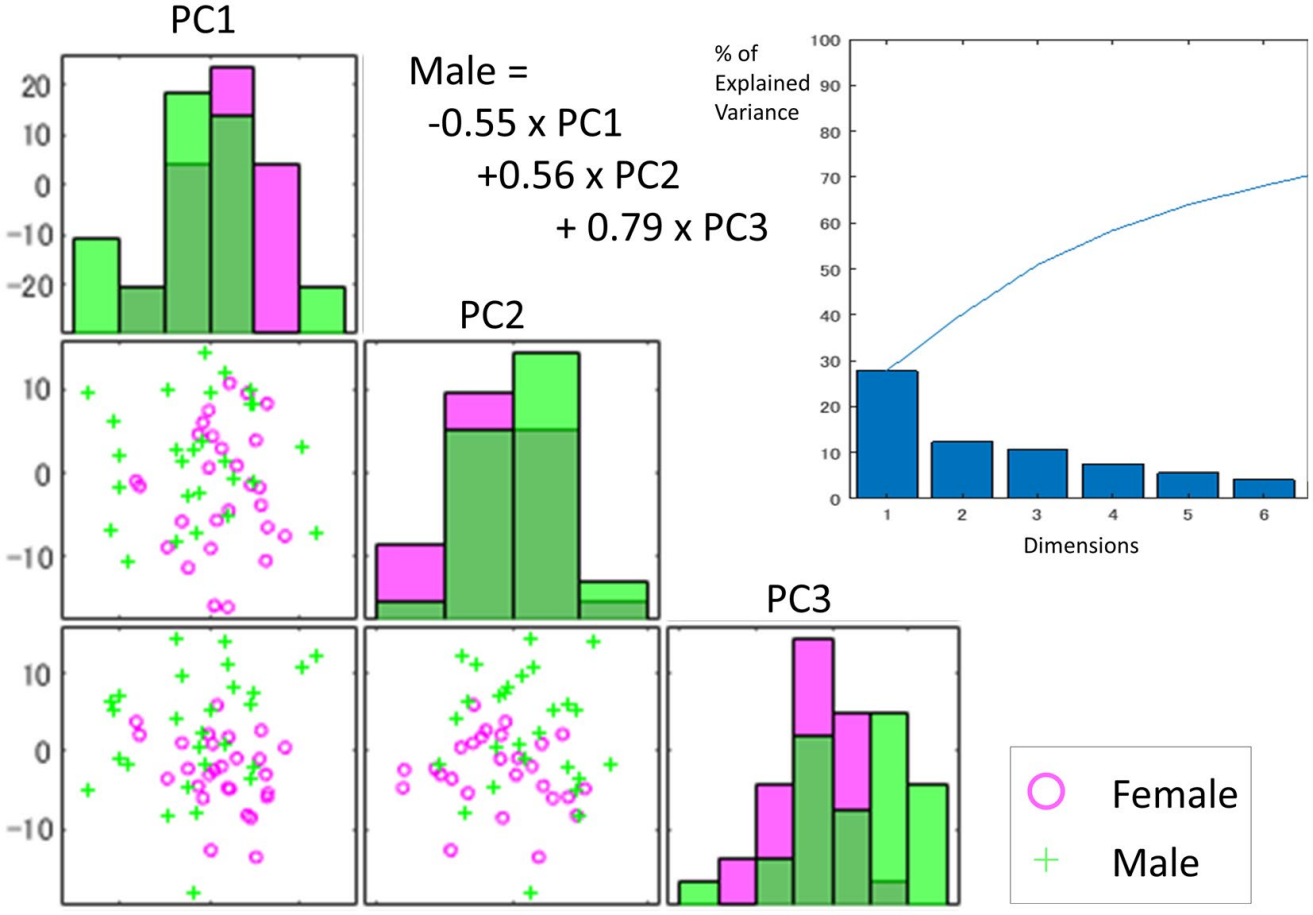
Scatter plot of principal components (PC) 1, 2, and 3 for the female and male groups. The top-right shows a scree plot (the x-axis shows the number of PCs and the y-axis shows the % of explained variance of the total variance). The green plus mark denotes male subjects, and the pink circle mark denotes female subjects. The function represents the results of discriminant analysis using PCs.

**Figure 5:**
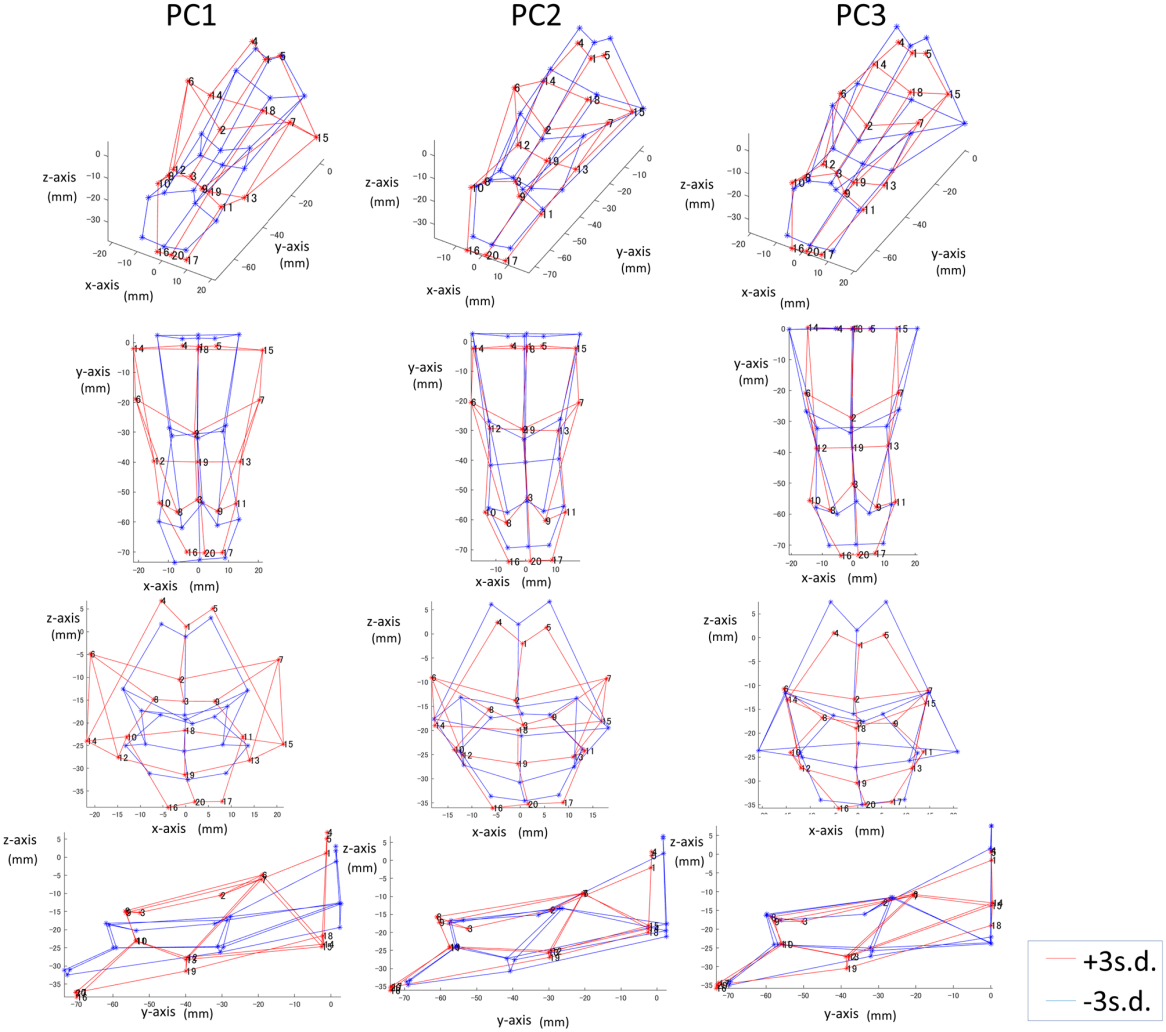
Visualization of the principal components (PCs) 1, 2, and 3. Red lines shows the virtual shape located at the + 3 standard deviation (SD) of each PC. Blue denotes − 3 SD. PC1 indicates the ratio of the vertical length in comparison to the anteroposterior length and the horizontal size of the upper limit of the oropharynx. A greater PC1 indicates a vertically shorter airway in comparison to the anteroposterior and horizontal dimensions. PC2 indicates the width of the oropharyngeal wall and the vertical positions of the narrowest point of the oropharynx. A greater value indicates a greater oropharyngeal wall width and vertically higher position in comparison to a smaller PC2. PC3 indicates the horizontal size of the upper limit of the oropharynx in comparison to the total airway shape. A greater PC3 value indicates a smaller upper limit of the oropharynx in comparison to a smaller PC3, indicating an inversed triangle shape when viewed from the front.

The variances in the first three PCs (PC1, PC2, and PC3) were greater than 10% of the total variance (Fig. 4). PC1 indicates the ratio of the vertical length to the anteroposterior length and horizontal size of the upper limit of the oropharynx. PC2 indicates the width of the oropharyngeal wall and vertical position of the narrowest point of the oropharynx (Fig. 5). PC3 indicates the horizontal size of the upper limit of the oropharynx compared to the total airway shape. Discriminant analysis showed that the airway shape of males was characterized by a smaller PC1 (standardized coefficients = − 0.55), greater PC2 (− 0.55), and greater PC3 (+ 0.79) (Wilks’ lambda = 0.56, Chi-square = 31.5, p < 0.001). That is, in comparison to females, males had a vertically longer airway in comparison to the anteroposterior and horizontal dimensions (PC1), a greater width of the oropharyngeal wall and vertically higher positions of the narrowest point of the oropharynx (PC2), and a smaller upper limit of the oropharynx in comparison to the total shape (PC3). The findings in PC2 corresponded to the distance results (i.e., the distance below the narrowest point from the bottom of the hypopharynx was greater in males). The correctly classified percentage for the total sample was 74.1%.

## Discussion

To our knowledge, this is the first study to determine sex differences in 3D airway shape in skeletal 1 subjects. In summary, it was found that the volume of the airway showed no significant differences (Table 4), but males tended to show a long and straight airway shape, while females had an inverted triangular airway shape from the frontal view with a shorter vertical length from the narrowest point of the airway to the bottom of the hypopharynx (Fig. 3 and Table 5).

In the clinical setting, we confirmed the abnormality in a patient in comparison with normal samples. A relative increase in airway volume after mandibular set-forward, maxillary expansion, or orthodontic treatment can be suggested as a possible treatment option for patients with OSA^2,3,9^. Although these are not the first choice of treatment, the measurement of the airway volume and shape and comparison of these values to a normative mean would be the first step in treatment planning. Indeed, the criteria used in decision making in relation to mandibular advancement in OSA are now solely based on subjective judgment. If we could correct the data on the relationships between advancement and increment of the volumes^10^ using this normative data, comparison with a normative mean would be helpful for deciding the amount of advancement.

A previous study showed that the volumes varied among skeletal classifications I, II, and III^11^ (i.e., Class II had a smaller airway volume than Class I and III). However, when comparing the OSA and non-OSA groups, a previous report showed that there were no significant differences in airway volumes. In contrast, when subjects were classified according to their total apnea–hypopnea index, the severe OSA group had a longer airway length relative to the control group^12^, which indicates the importance of shape analysis. Further research regarding the shapes of patients with OSA is needed, using the present study as the basis for comparison.

Sex differences in the frequency of OSA have been considered to be primarily a disease associated with male sex; however, it is now being shown that different indices should be used to determine this in men and women. That is, women are more symptomatic than men, even with lower apnea–hypopnea index scores, and the actual sex difference is unknown^13,14^. The present study showed that there were no significant differences in airway volume; however, differences in shape were observed. Further research, including studies of the relationship between OSA and airway shape in both sexes, is important.

The present study has some limitations. The control group did not include patients with normal occlusion. Considering the basic principle of minimizing unnecessary radiation exposure, it is impossible to obtain CBCT images of a subject with perfectly normal occlusion without any complications. Similarly, it is difficult to determine the influence of posture because performing CBCT multiple times in the same participant is not permissible. Although we have tried to use the standardized protocol to perform CBCT, body posture inevitably influences the soft tissues.

OSA is a complex, multifactorial disease with several contributing factors, including airway morphology, muscle tonicity, aging, and sex. Thus, a comprehensive analysis is necessary to understand the interplay of various factors in analyzing and making treatment plans for OSA. Furthermore, OSA can be diagnosed using polysomnography rather than the pharyngeal shape. Thus, the present shape analysis may not directly contribute to the diagnosis or treatment plans in clinical practice.

However, the proposed method enables us to conduct a multidimensional analysis to clarify the future development of OSA with other possible factors. Our study used a geometric morphometric analysis, which offers a detailed 3D understanding of airway morphology. Our findings demonstrated distinct differences between the sexes, highlighting the significance of studying airway morphology in greater detail. Geometric morphometrics is a well-established method for assessing biological shape variations among populations, and its application in airway analyses can provide orthodontists with a comprehensive understanding of OSA. Thus, we believe that investigating airway morphology complements our understanding of OSA and other contributing factors. In the future, we intend to analyze the airway morphology in several malocclusion, non-OSA, and OSA patients, contributing to better orthodontic practices and the possible clarification of OSA development.

## Conclusion

- Normative data for 3D measurements of the upper airway in Japanese adults were established by sex, which allows clinicians to evaluate how the 3D characteristics of a patient's airway differ from the normative range.
- No significant sex differences were found in the volume of the airway, but males tended to show a long and straight airway shape, while females had an inverted triangular airway shape from the frontal view with a shorter vertical length from the narrowest point of the airway to the bottom of the hypopharynx.

## Data Availability

The data produced and analyzed in this study are available from the corresponding author upon reasonable request.

## References

[1] Neelapu BC, Kharbanda OP, Sardana HK (2017). Craniofacial and upper airway morphology in adult obstructive sleep apnea patients: A systematic review and meta-analysis of cephalometric studies. Sleep Med. Rev..

[2] Sahoo NK, Agarwal SS, Datana S, Bhandari SK (2021). Quantifying upper airway changes following mandibular orthognathic surgery. J. Craniofac. Surg..

[3] Lee WY, Park YW, Kwon KJ, Kim SG (2016). Change of the airway space in mandibular prognathism after bimaxillary surgery involving maxillary posterior impaction. Maxillofac. Plast. Reconstr. Surg..

[4] Zimmerman JN, Lee J, Pliska BT (2017). Reliability of upper pharyngeal airway assessment using dental CBCT: A systematic review. Eur. J. Orthod..

[5] Hatcher DC (2012). Cone beam computed tomography: Craniofacial and airway analysis. Dent. Clin. North Am..

[6] Xu C, Sin S, McDonough JM (2006). Computational fluid dynamics modeling of the upper airway of children with obstructive sleep apnea syndrome in steady flow. J. Biomech..

[7] Yoshikawa H, Tanikawa C, Ito S (2022). A three-dimensional cephalometric analysis of Japanese adults and its usefulness in orthognathic surgery: A retrospective study. J. Cranio-Maxillofac. Surg..

[8] Wada K (1977). A study of the individual growth of maxillofacial skeleton by means of lateral cephalometric roentgenograms. J. Osaka Univ. Dent. Sch..

[9] Stefanovic N, El H, Chenin DL, Glisic B, Palomo JM (2013). Three-dimensional pharyngeal airway changes in orthodontic patients treated with and without extractions. Orthod. Craniofac. Res..

[10] Bianchi A, Betti E, Badiali G, Ricotta F, Marchetti C, Tarsitano A (2015). 3D computed tomographic evaluation of the upper airway space of patients undergoing mandibular distraction osteogenesis for micrognathia. Acta Otorhinolaryngol. Ital..

[11] Zheng ZH, Yamaguchi T, Kurihara A, Li HF, Maki K (2014). Three-dimensional evaluation of upper airway in patients with different anteroposterior skeletal patterns. Orthod. Craniofac. Res..

[12] Kim EJ, Choi JH, Kim YS (2011). Upper airway changes in severe obstructive sleep apnea: Upper airway length and volumetric analyses using 3D MDCT. Acta Otolaryngol..

[13] Bonsignore MR, Saaresranta T, Riha RL (2019). Sex differences in obstructive sleep apnoea. Eur. Respir. Rev..

[14] Won CHJ, Reid M, Sofer T (2020). Sex differences in obstructive sleep apnea phenotypes, the multi-ethnic study of atherosclerosis. Sleep.

